# Combined effects of conditional cash transfer program and environmental health interventions on diarrhea and malnutrition morbidity in children less than five years of age in Brazil, 2006–2016

**DOI:** 10.1371/journal.pone.0248676

**Published:** 2021-03-30

**Authors:** Anelise Andrade de Souza, Sueli Aparecida Mingoti, Rômulo Paes-Sousa, Léo Heller

**Affiliations:** 1 Instituto René Rachou, Fiocruz Minas, Belo Horizonte, Minas Gerais, Brazil; 2 Department of Statistics, Federal University of Minas Gerais, Belo Horizonte, Minas Gerais, Brazil; Universidade Federal de Pelotas, BRAZIL

## Abstract

**Introduction:**

Governmental measures aiming at social protection, with components of disease control, have potential positive impacts in the nutritional and health outcomes of the beneficiaries. The concomitant presence of these measures with environmental sanitation interventions may increase their positive effect. The context of simultaneous improvement of social protection and environmental sanitation is found in Brazil since 2007 and an assessment of the combined effects of both programs has not been performed so far.

**Objective:**

To evaluate whether interaction effects between improvement of access to water, sanitation and solid waste collection with the Bolsa Família Program [PBF] were related to better responses in the reduction of morbidity due to diarrhea and malnutrition in children less than five years of age, acknowledging the positive results of these improved conditions and the PBF separately in coping with these diseases.

**Methods:**

Descriptive and inferential analyses were performed through Generalized Linear Models of the Negative Binomial type of fixed effects, with and without addition of zeros. Interaction models were inserted in order to evaluate the outcomes when the two public policies of interest in the current study were present simultaneously in the municipalities.

**Results:**

Interaction with negative effect when a concomitantly high municipal coverage of the Bolsa Família Program and adequate access to sanitation and solid waste collection were present. In contrast, regardless of municipal coverage by the PBF, the simultaneous presence of water and sanitation (0.028% / 0.019%); water and solid waste collection (0.033% / 0.014%); sanitation and solid waste collection (0.018% / 0.021%), all resulted in a positive effect, with a decrease in the average morbidity rates for both diseases.

**Conclusion:**

Investments aimed at universalizing water, sanitation and solid waste collection services should be priorities, aiming at reducing the incidence of morbidity due to malnutrition and diarrhea and preventing deaths from these poverty-related diseases.

## Introduction

Diarrhea and diseases associated with malnutrition are among the main causes of morbidity and mortality in children less than five years of age in developing countries [1–11]. In 2015, 5.9 million deaths of children under five occurred due to infectious diseases such as diarrhea, pneumonia, malaria, meningitis, tetanus and measles, with diarrhea and pneumonia being the biggest cause of death in this age group [1]. Regarding malnutrition, globally, one in three children under the age of five does not grow properly due to malnutrition, considering its visible forms. Among the main factors for preventing malnutrition, adequate food intake in quantity and quality, food security adequate access to drinking water, adequate sanitation and solid waste collection are essential [5]. In view of this, it is relevant for the governments to prioritize programs for social protection and environmental health, geared toward better nutritional and health outcomes for the population.

The Bolsa Família Program (PBF) was created in 2003 in Brazil. Its main objective is poverty reduction, to be achieved by working along three axes [12, 13]. The first axis corresponds to the direct transfer of income to poor or extremely poor families, with benefit values varying according to the socioeconomic status of families and their family composition [14]. The second axis corresponds to the expansion of access to public services that represent basic rights in the areas of health, education and social assistance, through the conditionalities of the Program. The objective of this axis is to allow families to break the intergenerational cycle of poverty reproduction. Finally, the third axis corresponds to the coordination of the PBF with other governmental actions and programs from all spheres (local, state and federal), in order to support families in overcoming situations of vulnerability and poverty [13].

At the same time, adequate conditions for access to services for water, sanitation and solid waste collection are considered as effective, lower-cost public health interventions for reducing cases of diarrhea and other water-related diseases, especially in developing countries [15, 16]. Brazil adopted the National Basic Sanitation Plan (PLANSAB) in 2007 [17], which determined new scenarios for the sector in the country and presented the possibility of almost universal coverage for the entire population of access to water, sanitation and solid waste collection by 2033. However, in general, although the current legislation highlights solutions for the universalization of these services, major deficiencies still prevail in the country, with the poorest populations being the most left behind [18].

In light of the above, although studies point to the positive health outcomes of the PBF itself [19–33] and of interventions related to environmental health [34–49], as well as to the effects of the PBF combined with other health policies and programs [27, 50–53], none have so far evaluated the joint action of these two public policies in an interaction model, which is the objective of the current study.

## Methods

This research uses an ecological design, with exploratory and analytical approaches, assessing the temporal trend of the rates of health events in different population groups (through the exploratory study) and the association between the average level of exposure and the rates of the events between different population groups, over time (through the analytical study). In the current study, data from 3,467 Brazilian municipalities were compiled each year for the period 2006–2016. The choice of this time frame was due to the information of interest in the current study, mainly that related to the PBF, are available on a consolidated basis only from the year 2006. In addition, this time frame allowed to assess the joint effects of the two public policies, of environmental sanitation and the PBF, since in the year of 2007 environmental sanitation was regulated by means of Law 11.455 [17], establishing its national guidelines. Two thousand and 16 was the last year with available data related to the outcome variables of interest, at the time of data collection. The municipalities, located throughout different regions of the country, were kept the same in all 11 years. Thus, there is a delineation in balanced panel data, with the municipality being the unit of analysis and the grand total of observations equal to 38,137.

We selected 3,467 out of the 5,560 Brazilian municipalities existing in the first year of evaluation of the study. The selection criteria was that a municipality, to be included, needed to present all the following data: (i) adequacy of vital statistics data [54] (ii) municipal annual data of hospitalization for diarrhea and malnutrition for children less than five years of age; (iii) annual data on coverage of the total population by the PBF; (iv) annual data on coverage of the target population by the PBF; (v) coverage data for water, sanitation and solid waste collection services for the years 2000 and 2010, due to this information being available only in the censuses carried out in these years in the country.

After the selection of the municipalities, the variables were collected, using public information systems, that could respond to the study hypothesis, that is, that better conditions of access to water, sanitation and solid waste collection, simultaneously present in municipalities with high coverage by the PBF, would result in lower morbidity rates due to diarrhea and malnutrition. Table 1 presents the variables available in the information systems that generated the dependent, independent and covariate variables used in the present study (Table 1). We assumed linear interpolation and extrapolation to predict the annual values of the variables that were not available, with the exception of the income variable.

**Table 1:** Variables available in the information systems and availability period.

Variables	Data source/information system	Period
Hospitalization for diarrhea^1^ (A00 –A04 and A06 –A09)^2^ and for malnutrition (E40 –E46)^2^ and number of children under the age of five	Hospital Information System (SIH) / Informatics Department of the Unified Health System (SUS) (DATASUS)	Years 2006 to 2016
Beneficiary families of the Bolsa Família Program (PBF)	Social Information Matrix (MIS) / Information Evaluation and Management Service (SAGI)	Years 2006 to 2016
Average size of beneficiary families	MIS / SAGI	Years 2007 and 2010
Families eligible for the Bolsa Família Program (PBF)	MIS / SAGI	Years 2006 to 2016
Number of households with coverage for water and sanitation services and number of municipal households	CENSO / IBGE	Years 2000 and 2010^3^
Population exposed to solid waste collection and total municipal population	CENSO / IBGE	Years 2000 and 2010^3^
Per capita monthly income	CENSO/IBGE	Years 2000 and 2010^3^
Proportion of individuals without basic literacy among the population aged 15 and over	CENSO / IBGE	Years 2000 and 2010^3^
Urbanization rate	IBGE	Years 2000 and 2010^3^
Population served by primary care related to the Family Health Strategy (ESF) and total municipal population	Primary Care Information System (SIAB) / DATASUS	Years 2006 to 2016

^1^Only categories related to hospitalization due to diarrhea were also chosen, which were also classified as Diseases Related to Inadequate Environmental Sanitation (DRSAI) [96].

^2^International Statistical Classification Codes for Diseases and Health-Related Problems—10th revision (ICD-10).

^3^For variables with information only for the years related to the censuses, 2000 and 2010, interpolation (2006 to 2009) and linear extrapolation (2011 to 2016) methods were applied. For the income variable, due to its non-linear behavior [97], the variation of municipal Gross Domestic Product (GDP) was used to predict the variation of municipal income and after this procedure, their values were corrected according to the Consumer Price Index Broad (IPCA).

From the database, the values of the dependent variables were estimated, through the ratio between: the number of admissions notified in the Hospital Information System (SIH), based on the Hospitalization Authorization (AIH), and the total number of children living in the municipality of the same age group and of the same year, multiplied by 10,000, thus using a traditional measure for rare disease morbidity rates.

The following independent variables were included in the study: (i) coverage of the total population by the PBF, obtained by multiplying the number of beneficiary families and average family size, divided by the total municipal population; (ii) coverage of the target population by the PBF, calculated as the ratio between the number of families benefiting from the Program in a municipality and the number of eligible families in the same municipality according to the PBF criteria (families considered "extremely poor", with a monthly income per capita of up to $ 16.9 dollars per person or "poor", with a monthly income per capita of $ 16,9 to $ 33.8 dollars); (iii) coverage of the total population of access to water, calculated as the ratio between the number of households with access to the water network, well or cistern and the number of municipal households; (iv) coverage of the total population of access to sanitation, calculated as the ratio between the number of households with access to the sanitation network or septic tank and the number of municipal households; (v) coverage of the total population of solid waste collection, calculated as the ratio between the population exposed to solid waste collection and total municipal population.

The following covariates, considered possible determinants of morbidity processes due to diarrhea and malnutrition, were used as confounding variables: (i) monthly income *per capita* of the municipal population (in Brazilian Reais), corrected by inflation for the period; (ii) literacy of the population 15 years of age and older; (iii) coverage of the total population of the municipality by the Family Health Strategy (ESF), calculated as the ratio between the population assisted by primary care related to the ESF and the total municipal population; (iv) municipal urbanization rate.

Finally, the variables population size, years and Brazilian regions were inserted in the regression models.

Morbidity rates were expressed per ten thousand inhabitants and the other variables, also used continuously, expressed in percentage, except for income values that were used through their medians.

Subsequent to the collection of information and calculation of the variable values, descriptive statistical analyses were performed, using mean, median and standard deviation values. Inferential statistical analyses was performed using the Generalized Linear Model with fixed effects considering the Poisson and the Negative Binomial distributions, without and with zero inflation. The statistical analysis with the adjustment for excess of zeros was employed in order to verify the robustness and consistency of the analyses. In this modeling it is possible to adjust a regression model which takes into account the frequency of zeros generated by the Poisson and the Negative Binomial distributions, as well as the frequency of zeros that is generated by another distribution related to the excess of zero. Although, there are many distributions that can be used with this purpose, the logistic distribution is very common. Therefore, in the adjustment of zero inflated models two probability distributions are combined to generate estimates of the number of cases (and rates) for each sample unit considering the respective values of the explanatory variables [55, 56]. In the univariate analyses, a significance level of 25% (p-value = 0.25) [57] was used in the selection of variables to make up the multivariate regression models [58]. In these, the significance level of 5% (p<0.05) was used to maintain the variables in the final models. Finally, in the interaction models between the environmental health and PBF variables, the significance level of 10% (p<0.10) [59] was used. The criteria used in the choices of regression models were: (i) Akaike Information Criterion (AIC); (ii) Bayesian Information Criterion (BIC); (iii) better adjustment of deviations (*Deviance* and *Pearson*); (iv) better ability to predict hospitalization frequencies; (v) possibility of evaluating the interactions of interest of the current study; (vi) the performance in the residual analysis.

*Microsoft Office Excel 2010* software was used for database construction and *software R (*version 3.0.2*)* 2013 (*The R Foundation for Statistical Computing*) in descriptive and inference analyses. The MASS [60] package of the *software R* was used to adjust the Poisson regression Generalized Linear Model and Negative Binomial, with no zero inflation. For both models, for adjustment by excess zeros, the pscl (*Political Science Computational Laboratory*) package [61] was used.

This study was conducted exclusively with secondary and aggregated data, publicly accessible and in accordance with resolutions of the National Health Council No. 466/2012 [62] and No. 510/2016 [63], exempt from evaluation by the Research Ethics Committee.

## Results

We selected 3,467 municipalities, corresponding to 62.3% of the total number of Brazilian municipalities in 2006 and 38,137 observations evaluated through panel data. Table 2 shows the result of descriptive statistics by year of analysis, according to dependent, independent and covariate variables of interest. The percentage change for the years 2006 and 2016 showed a reduction in morbidity rates by 38.46% and 51.35% for malnutrition and diarrhea, respectively.

**Table 2:** Descriptive measures of morbidity rates by years of study and municipalities selected—Brazil (N = 3467).

	2006 (min-max)	2007 (min-max)	2008 (min-max)	2009 (min-max)	2010 (min-max)	2011 (min-max)	2012 (min-max)	2013 (min-max)	2014 (min-max)	2015 (min-max)	2016 (min-max)	Percentage Change 2006–2016 (CI)
Morbidity												
Due to malnutrition	5.2 ±13.5 0.0–239.3	4.3 ±12.5 0.0–303.0	4.50 ±12.1 0.0–243.9	4.1 ±11.5 0.0–152.9	3.9 ±11.9 0.0–201.1	3.5 ±10.9 0.0–262.8	3.6 ±11.7 0.0–300.3	3.3 ±10.6 0.0–188.7	3.6 ±10.4 0.0–128.2	3.2 ±10.2 0.0–141.5	3.2 ±9.9 0.0–154.3	- 38.46%±11.4 (38.08–38.83)
Due to diarrhea	193.0 ±196.4 0.0–1723.7	136.2 ±148.3 0.0–1401.6	155.5 ±167.7 0.0–1270.6	132.7 ±155.0 0.0–1327.8	158.0 ±187.3 0.0–2396.9	95.5 ±128.3) 0.0–1106.4	111.3 ±147.4 0.0–1562.5	98.9 ±141.7 0.0–1450.4	106.3 ±149.2 0.0–1371.2	80.9 ±123.5 0.0–1146.9	93.9 ±148.0 0.0–1410.7	- 51.35%±158.7 (46.06–56.63)
Proportion of coverage of the total population by PBF	31.2% ±18.6 1.2–100.0	31.1% ±19.1 0.7–100.0	28.2% ±18.0 0.2–100.0	30.8% ±18.5 0.2–81.9	31.2% ±19.0 0.5–83.9	32.4% ±20.6 0.4–89.3	33.2% ±21.2 0.7–96.5	32.4% ±21.0 0.3–93.4	32.1% ±21.8 0.4–97.4	30.5% ±20.6 0.5–96.2	29.3% ±20.8 0.1–99.9	- 6.09%±20.0 (5.42–6.75)
Proportion of coverage of the target population by the PBF	87.2% ±15.9 10.8–100.0	86.8% ±15.2 12.5–100.0	83.1% ±16.5 10.0–100.0	90.8% ±12.9 6.4–100.0	92.3% ±12.3 18.6–100.0	93.2% ±13.1 8.8–100.0	94.4% ±12.1 12.3–100.0	94.3% ±12.7 5.3–100.0	91.9% ±14.6 11.4–100.0	91.7% ±15.1 8.8–100.0	71.7% ±34.0 0.2–100.0	- 17.77%±18.0 (17.17–18.36)
Proportion of sanitation coverage	39.3% ±29.7 0.1–97.5	40.0% ±29.7 0.1–97.2	40.8% ±29.8 0.1–96.9	41.5% ±30.0 0.1–96.6	42.2% ±30.3 0.0–96.7	43.2% ±30.6 0.0–100.0	44.3% ±30.9 0.0–100.0	45.5% ±31.2 0.0–100.0	46.7% ±31.6 0.0–100.0	48.0% ±31.9 0.0–100.0	49.4% ±32.3 0.0–100.0	25.70%±30.8 (24.67–26.72)
Proportion of water coverage	87.3% ±14.2 4.3–100.0	87.2% ±14.0 3.8–100.0	87.1% ±13.9 3.3–100.0	87.0% ±13.9 2.6–100.0	86.9% ±14.0 1.8–100.0	86.8% ±14.1 1.5–100.0	86.7% ±14.2 1.2–100.0	86.6% ±14.3 0.9–100.0	86.5% ±14.5 0.8–100.0	86.3% ±14.6 0.6–100.0	86.2% ±14.8 0.5–100.0	- 1.26%±14.22 (0.78–1.73)
Proportion of solid waste collection	63.3% ±22.3 4.1–98.8	64.8% ±21.7 4.7–98.8	66.2% ±21.3 5.4–98.9	67.7% ±20.9 6.0–98.9	69.2% ±20.5 6.6–98.9	71.0% ±20.3 7.3–100.0	73.0% ±20.1 8.2–100.0	74.9% ±19.8 8.8–100.0	76.8% ±19.6 9.2–100.0	78.7% ±19.2 9.5–100.0	80.6% ±18.9 9.9–100.0	27.33%±21.1 (26.62–28.03)
Proportion of coverage of the total population by the FHS	72.2% ±31.1 0.0–100.0	74.9% ±29.7 0.0–100.0	79.7% ±29.1 0.0–100.0	80.5% ±28.3 0.0–100.0	82.0% ±27.6 0.0–100.0	83.0% ±27.1 0.0–100.0	83.3% ±26.8 0.0–100.0	84.3% ±25.3 0.0–100.0	86.2% ±22.8 0.0–100.0	88.3% ±20.9 0.0–100.0	88.5% ±20.9 0.0–100.0	22.58%±26.9 (21.68–23.47)
Urbanization rate (%)	62.6% ±21.5 4.9–99.7	62.9% ±21.3 5.0–99.6	63.3% ±21.1 5.1–99.6	63.6% ±21.0 5.2–99.5	64.0% ±20.9 5.4–99.5	64.5% ±20.8 5.5–100.0	65.1% ±20.8 5.6–100.0	65.7% ±20.8 5.8–100.0	66.4% ±20.8 5.9–100.0	67.0% ±20.8 6.1–100.0	67.7% ±20.9 6.3–100.0	8.15%±21.0 (7.45–8.84)
Per capita monthly income in reais (R$)*	353.9 ±208.5 42.7–1938.0	368.2 ±218.3 44.3–1835.7	365.9 ±216.9 44.5–1810.0	357.8 ±2 10.0 26.1–1741.5	514.4 ±259.2 124.4–2127.7	370.8 ±217.8 29.1–1812.4	369.1 ±217.0 28.3–1801.8	376.2 ±222.2 27.4–1849.1	379.5 ±224.1 27.2–1895.4	396.0 ±234.3 27.6–1973.4	383.0 ±226.6 26.3–1872.2	8.22%±227.3 (0.66–15.78)
Proportion literate individuals	71.0% ±11.0 23.8–93.5	70.9% ±10.6 18.5–93.3	70.0% ±9.9 29.9–92.5	68.5% ±10.2 27.1–92.1	67.0% ±10.2 29.8–91.9	74.6% ±7.0 29.8–91.9	73.0% ±7.4 49.1–92.7	71.4% ±7.7 46.8–92.2	69.6% ±8.1 42.6–91.7	67.7% ±8.6 37.7–91.1	65.6% ±9.1 32.3–90.5	- 7.61%±9.5 (7.29–7.92)

Data refer to the mean and (standard deviation). For income*, the median was considered. Causes of morbidity in children less than five years old are defined according to the International Classification of Diseases (CID-10), 10th revision: diarrheal diseases (A00, A01, A02, A03, A04, A06-08) and malnutrition diseases (E40—E46). Morbidity rates are shown in the table for every ten thousand children up to five years old. N = number of municipalities. PBF = Bolsa Família Program. ESF = Family Health Strategy.

Regarding the independent variables, the percentage change between the first and last year of the study showed a reduction in the proportion of coverage of the total municipal population by the PBF, the population targeted by the PBF and municipal coverage of access to water. Specifically, for the coverage of the target population by the PBF, 2016 was the only year, of all years evaluated in this study, with coverage values below 80% (71.7%). The year 2012, in contrast, was the year with the highest average coverage, both for the target population (94.4%) as well as for the total municipal population by the Program (33.2%). At the same time, although there was a decrease in the percentages of municipal coverage of access to water, their mean values remained high (86.2%) and very close to those prevalent in the first year of evaluation of the present study (87.3%). In contrast to the reduction of the independent variables mentioned above, there was an average increase in the access of the municipal population to sanitation services (39.3% to 49.4%), in addition to a substantial average increase in the proportion of municipal coverage of access to solid waste collection (63.3% to 80.6%). In relation to the covariates evaluated, they presented higher mean values in 2016, compared to 2006: coverage of the municipal population to the ESF (72.2% to 88.5%), urbanization rate (62.6% to 67.7%) and monthly income *per capita* adjusted (R$ 353.9 to R$ 383.0). In contrast, the covariate literacy percentage of the population aged 15 years or older showed a slight decline (71.0% to 65.6%).

Table 3 presents independent and control variables categorized according to IRR value and the meaning of the IRR values generated in the regression models. Tables 4 and 5 present estimates of incidence rate ratios (IRR)—which represent the multiplier effect on the mean of the response variable resulting from the change of a unit of measurement of the explanatory variable—and 95% confidence interval, resulting from the adjustments of the negative binomial regression models, that best fit the data, of fixed effects with and without zero inflation, for the mean morbidity rates due to malnutrition and diarrhea, respectively, related to the total municipal coverage by the PBF and environmental health variables, controlling the health covariates of the study. For the malnutrition outcome (Table 4), the following variables for the models without and with zero inflation were statistically significant: (i) coverage of the total population by the PBF; (ii) access to water; (iii) access to sanitation; (iv) access to solid waste collection; (v) literacy of the population aged 15 years or older; (vi) population; (vi) monthly income *per capita*; (vii) coverage of the total population by the ESF, in addition to the variables related to the years 2007 to 2016. For the diarrhea outcome (Table 5), the variables for the models without and with zero inflation were significant: (i) coverage of the total population by the PBF; (ii) access to water; (iii) access to sanitation; (iv) access to solid waste collection; (v) literacy of the population aged 15 years or older; (vi) urbanization rate; (vii) monthly income *per capita*, in addition to the variables related to the years 2007 to 2016. The results show a negative effect, with an increase in the mean morbidity rates for both diseases, when the municipalities have higher coverage by the PBF and higher coverage of access to water. In turn, municipalities with high coverage of access to sanitation services and solid waste collection showed as a result the decrease in the average morbidity rates due to malnutrition and diarrhea, and thus, positive effect. Regarding the covariates, for the morbidity due to malnutrition outcome, coverage by the ESF, literacy of the population aged 15 years or more and population size had a positive effect, as opposed to monthly income *per capita*. For the morbidity due to diarrhea outcome, higher literacy percentages were associated with lower morbidity rates due to this disease, as well as higher rates of urbanization and monthly income *per capita*, with higher morbidity rates due to diarrhea. For both outcomes, the years 2007 to 2016 presented lower morbidity rates when compared to 2006. Table 6 shows the adjusted generalized variance inflation factors (AGVIF) for the negative binomial generalized regression models without zero inflation (for malnutrition and diarrhea). The AGVIF values are smaller than 3 for both models indicating no presence of substantial multicollinearity. The common reference value used when analysing adjusted AGVIF values was 5 [64]. No autocorrelation was detected in the residuals of the models presented in this paper, nor when analysing the residuals considering each year separately, nor when the residuals of all years were combined in a single data set.

**Table 3:** Independent and control variables categorized according to IRR value.

	IRR* > 1	IRR* < 1
Malnutrition	Bolsa Família Program (PBF) total	**-**
	Access to water	**-**
	^**-**^	Access to sanitation
	**-**	Access solid waste collection
	**-**	Literacy population 15 years or older
	Per capita income	
	**-**	Population
	**-**	Coverage ESF
	**-**	Years 2007–2016
Diarrhea	Bolsa Família Program (PBF) total	-
	Access to water	-
	-	Access to sanitation
	-	Access solid waste collection
	-	Literacy population 15 years or older
	Per capita income	-
	-	Urbanization rate
	-	Years 2007–2016

* Ratio for incidence rates.

IRR* > 1 (it means that the increase in municipal coverage by the variable that generated the IRR results in an increase in the average rates of morbidity due to diarrhea and / or malnutrition).

IRR* < 1 (it means that the increase in municipal coverage by the variable that generated the IRR results in a decrease in the average rates of morbidity due to diarrhea and / or malnutrition).

**Table 4:** IRR results—fixed-effects Negative Binomial (NB) regression model to assess morbidity due to malnutrition in children less than five years old.

	NB regression model without zero inflation	NB regression model with zero inflation
	IRR* (95% CI) p-value	IRR* (CI) p-value
Bolsa Família Program (PBF)		
PBF total	1.0057 (1.0034, 1.0081) 1.23e-06	1.0043 (1.0017, 1.0069) 0.00
Environmental Health		
Access to water	1.0059 (1.0038, 1.0080) 1.93e-08	1.0097 (1.0071, 1.0123) 2.05e-13
Access to sanitation	0.9972 (0.9962, 0.9981) 9.64e-09	0.9943 (0.9931, 0.9954) < 2e-16
Access solid waste collection	0.9963 (0.9947, 0.9980) 2.32e-05	0.9964 (0.9947, 0.9982) < 2e-16
ESF	0.99913 (0.9982, 1.0000) 0.05	-
Literacy population 15 years or older	0.9804 (0.9770, 0.9839) < 2e-16	0.9766 (0.9725, 0.9806) 5.24e-05
Per capita income	1.2960 (1.1983, 1.4017) 6.37e-11	1.3705 (1.2655, 1.4841) 8.84e-05
Population	0.9591 (0.9372, 0.9817) 0.00	0.8659 (0.8405, 0.8921) < 2e-16
Year 2007	0.8432 (0.7710, 0.9221) 0.00	0.8481 (0.7672, 0.9376) 0.00
Year 2008	0.9021 (0.8246, 0.9869) 0.02	0.9010 (0.8163, 0.9945) 0.03
Year 2009	0.7972 (0.7274, 0.8735) 1.35e-06	0.8105 (0.7309, 0.8988) 6.86e-05
Year 2010	0.6869 (0.6218, 0.7587) 9.78e-14	0.6870 (0.6150, 0.7674) 2.89e-11
Year 2011	0.8090 (0.7358, 0.8895) 1.23e-05	0.8875 (0.7955, 0.9900) 0.03
Year 2012	0.7631 (0.6935, 0.8396) 3.03e-08	0.8316 (0.7446, 0.9288) 0.0
Year 2013	0.7024 (0.6375, 0.7738) 8.91–13	0.7850 (0.7022, 0.8776) 2.10e-05
Year 2014	0.7437 (0.6747, 0.8197) 2.26–09	0.8238 (0.7377, 0.9199) 0.00
Year 2015	0.6282 (0.5681, 0.6946) < 2e-16	0.6854 (0.6118, 0.7678) 7.15e-11
Year 2016	0.6441 (0.5818, 0.7129) < 2e-16	0.7123 (0.6348, 0.7992) 7.68e-09
Log (theta)	..	< 2e-16

Model without zero inflation: AIC: 72641. BIC: 72812.3. 2 x loglik: -72601.3260. Zero inflation model: AIC: 72321.39. BIC: 72395.76. 2 x log-lik: -72240. Per capita income and Population in a logarithm scale.

*Ratio for incidence rates.

Note: CI (Confidence Interval). Model dependent variable: morbidity malnutrition. Sample size: 3467 cities observed along 11 years comprising a total of 38137 observations.

**Table 5:** IRR results—fixed-effects Negative Binomial (NB) regression model to assess morbidity due to diarrhea in children less than five years old.

	NB regression model without zero inflation	NB regression model with zero inflation
	IRR* (95% CI) p-value	IRR* (CI) p-value
Bolsa Família Program (PBF)		
PBF total	1.0196 (1.0183, 1.0209) < 2e-16	1.0192 (1.0179, 1.0205) < 2e-16
Environmental Health		
Access to water	1.0073 (1.0061, 1.0084) < 2e-16	1.0073 (1.0062, 1.0084) < 2e-16
Access to sanitation	0.9948 (0.9943, 0.9953) < 2e-16	0.9948 (0.9943, 0.9953) < 2e-16
Access solid waste collection	0.9916 (0.9904, 0.9927) < 2e-16	0.9924 (0.9913, 0.9936) < 2e-16
Literacy population 15 years or older	0.9898 (0.9879, 0.9917) < 2e-16	0.9894 (0.9875, 0.9913) < 2e-16
Per capita income	1.4519 (1.3891, 1.5174) < 2e-16	1.4332 (1.3712, 1.4980) < 2e-16
Urbanization rate	1.0072 (1.0062, 1.0081) < 2e-16	1.0068 (1.0058, 1.0078) < 2e-16
Year 2007	0.6972 (0.6619, 0.7344) < 2e-16	0.6979 (0.6628, 0.7349) < 2e-16
Year 2008	0.8568 (0.8134, 0.9025) 5.62e-09	0.8547 (0.8117, 0.9000) 2.63e-09
Year 2009	0.6885 (0.6533, 0.7256) < 2e-16	0.6873 (0.6523, 0.7241) < 2e-16
Year 2010	0.7049 (0.6665, 0.7456) < 2e-16	0.7059 (0.6676, 0.7664) < 2e-16
Year 2011	0.5103 (0.4838, 0.5383) < 2e-16	0.5120 (0.4854, 0.5400) < 2e-16
Year 2012	0.6016 (0.5704, 0.6346) < 2e-16	0.6037 (0.5724, 0.6367) < 2e-16
Year 2013	0.5243 (0.4969, 0.5533) < 2e-16	0.5267 (0.4992, 0.5558) < 2e-16
Year 2014	0.5760 (0.5455, 0.6082) < 2e-16	0.5777 (0.5468, 0.6095) < 2e-16
Year 2015	0.4370 (0.4133, 0.4620) < 2e-16	0.4387 (0.4149, 0.4638) < 2e-16
Year 2016	0.5238 (0.4950, 0.5543) < 2e-16	0.5259 (0.4970, 0.5665) < 2e-16

Model without zero inflation: AIC 269291. BIC: 269453.6. loglik: -269253.12800. Zero inflation model: AIC: 269172.8. BIC: 269240.37. 2 x loglik: -269126.8. Per capita income in a logarithm scale.

* Ratio of the incidence rates.

Note: CI (Confidence Interval). Model dependent variable: morbidity malnutrition. Sample size: 3467 cities observed along 11 years comprising a total of 38137 observations.

**Table 6:** Generalized Variance Inflation Factors (GVIF).

	GVIF	Df	AGVIF ^
**Malnutrition**			
PBF total	5.501254	1	2.345475
Access to water	2.075357	1	1.440610
Access to sanitation	2.096598	1	1.447963
Access solid waste collection	3.329224	1	1.824616
Literacy population 15 years or order	3.301881	1	1.817108
ESF	1.597700	1	1.264002
Per capita income	5.668062	1	2.380769
Population	1.693144	1	1.301209
Years	1.810010	10	1.030111
**Diarrhea**			
PBF total	5.193956	1	2.279025
Access to water	2.012644	1	1.418677
Access to sanitation	1.814025	1	1.346858
Access solid waste collection	4.248785	1	2.061258
Literacy population 15 years or order	2.534250	1	1.591933
Urbanization rate	3.091193	1	1.758179
Years	1.733984	10	1.027903

Note: DF: degrees of freedom. AGVIF is the adjusted Generalized variance inflation factor. It is defined as the value of GVIF to the power of (1/2 multiplied by DF).

Tables 7 and 8 show the IRR and 95% confidence intervals of the Negative Binomial regression models of fixed effects, with and without zero inflation, adjusted including interactions. They provide more details on the complex pattern of effects and interactions (p<0.10) of the PBF and environmental health interventions in the mean morbidity rates due to malnutrition and diarrhea, respectively. The objective was to evaluate the effect of each interaction separately. For this purpose, the interaction models were adjusted one by one. Therefore, the interaction term was different for each model. There were no problems in adjusting the models without zero inflation. However, due to the lack of convergence in the parameter estimation process, some interactions could not be evaluated in the adjusted models with zero inflation. All variables that were significant in the adjusted models without interaction remained in the models with interaction. In order not to increase the lengh of the article, only the results obtained for the terms of interaction of each model, are presented in Tables 7 and 8. The interactions related to the following terms and IRR were significant for the outcome (Table 7): (i) coverage by total PBF and access to sanitation; (ii) coverage by the total PBF and access to solid waste collection; (iii) access to water and sanitation; (iv) access to water and solid waste collection; (v) access to sanitation and solid waste collection. Similarly, interactions related to the following terms and IRR were significant for the outcome diarrhea (Table 8): (i) coverage by total PBF and access to sanitation; (ii) total PBF coverage and access to solid waste collection; (iii) access to water and sanitation; (iv) access to water and solid waste collection; (v) access to sanitation and solid waste collection.

**Table 7:** Results of the fixed-effects Negative Binomial (NB) regression model to assess the interaction to the outcome of morbidity due to malnutrition in children less than five years old.

	Regression model NB without zero inflation	NB regression model with zero inflation
	IRR* (CI) p-value	IRR* (CI) p-value
Interaction between: PBF total Access to sanitation	1.00018 (1.00014, 1.00022) < 2e-16	1.00020 (1.0001, 1.0003) 3.83e-08
Interaction between: PBF total Access solid waste collection	1.00035 (1.00029, 1.00040) < 2e-16	..
Interaction between: Access water Access to sanitation	0.99972 (0.99966, 0.99979) < 2e-16	..
Interaction between: Access water Access solid waste collection	0.99967 (0.99959, 0.99974) < 2e-16	..
Interaction between: Access to sanitation Access solid waste collection	0.99982 (0.99978, 0.99987) 6.07e-16	..

* Ratio for incidence rates.

Note: CI (Confidence Interval). Model dependent variable: malnutrition morbidity. Sample size: 3467 municipalities (38137 observations). ..interactions were not possible to be adjusted.

**Table 8:** Results of the fixed-effects Negative Binomial (NB) regression model to assess the interaction to the outcome of morbidity due to diarrhea in children less than five years old.

	Regression model NB without zero inflation	NB regression model with zero inflation
	IRR* (CI) p-value	IRR* (CI) p-value
Interaction between: PBF total Access to sanitation	1.00014 (1.00012, 1.00017) < 2e-16	1.00010 (1.0001, 1.0002) < 2e-16
Interaction between: PBF total Access solid waste collection	1.00016 (1.00013, 1.00019) < 2e-16	..
Interaction between: Access water Access to sanitation	0.99981 (0.99977, 0.99984) < 2e-16	..
Interaction between: Access water Access solid waste collection	0.99986 (0.99982, 0.99990) 8.24e-12	..
Interaction between: Access to sanitation Access solid waste collection	0.99979 (0.99976, 0.99981) < 2e-16	..

* Ratio for incidence rates.

Note: CI (Confidence Interval). Model dependent variable: diarrhea morbidity. Sample size: 3467 municipalities (38137 observations). ..interactions were not possible to be adjusted.

## Discussion

The results show that the average morbidity rates due to malnutrition and diarrhea in children less than five years of age decreased when compared to the years 2006 and 2016. However, high values of average morbidity rates due to diarrhea persisted. This trend has also been observed in other studies that evaluated Brazilian municipalities or states through hospitalization information [2, 3, 37, 65–67], which shows that these diseases, especially diarrhea, cannot be disregarded and should continue to be considered in Brazil as a public health problem.

Regarding the independent variables, between 2006 and 2016, a decrease in the total municipal coverage by the PBF as well as a decrease in the coverage of the target population by the Program and services for access to water were observed. Regarding the coverage of the total population by the PBF, the decrease in coverage may be an indication of improvements in the social conditions of the benefited municipalities and creation of development opportunities for beneficiary families [21, 68–71]. On the other hand, the decrease in the coverage of the target population by the PBF means that families eligible for the PBF increasingly had not received the benefit. Thus, the presence in Brazilian municipalities of a population without access to a minimum income and living in conditions of economic and social vulnerability can result in increased average morbidity rates and consequently mortality from malnutrition and diarrhea, diseases directly related to poverty [27, 72–74]. Finally, the decreasing values related to coverage of access to water services observed may result from the already high coverage prevalent in the first year of evaluation of the current study [73]. In contrast to these results, there was an increase in access coverage to solid waste collection and sanitation services. However, the increase verified for sanitation was not enough for the average national coverage to exceed 50%, demonstrating that the country is still far from the necessary process of universalization of this service [75–77].

At the same time, the covariate urbanization rate, the proportion of coverage by the ESF and monthly income *per capita* showed increases in percentages and coverage values when comparing the years 2006 and 2016. A possible explanation for this result is related to the increase in urbanization rates in the country, a movement that began in the 1930s which is still increasing, but at a reduced rate, and which has not resulted in better living conditions for most populations living in urban areas. Unbridled urbanization, without regulatory and control mechanisms, has enormous repercussions on the health of the population. Problems such as the insufficiency of basic services for access to water and sanitation, inadequate collection and disposal of solid waste, combined with precarious housing conditions, worsen conditions traditionally related to poverty. Poor populations concentrate most of the negative effects of urbanization, generating a situation of extreme inequality and environmental and health inequality [78–82]. On the other hand, the increase in municipal coverage by the ESF reflects its importance as a priority strategy for structuring Primary Health Care (PHC), serving as the main gateway to the Unified Health System (SUS) in the country. Investments in primary care through the ESF have brought many positive results, such as a reduction in the infant mortality rate, fewer hospital admissions potentially sensitive to PHC, greater equity, more access and continuity of care, and lower cost for the three governmental spheres [83, 84]. At the same time, the increase in monthly income *per capita*, resulting from including the presence of the PBF in all Brazilian municipalities, contributes to the advancement of the economic and social situation of the country, mainly at the local level, and has important impacts on poverty reduction and consequently on the reduction of food insecurity, thus improving the nutritional and health status of the population [21, 68–71, 83]. Regarding literacy, contrary to expectations [27, 85–87], the proportion of literate individuals in the population aged 15 years or older, showed a slight decline between the first and last year of analysis, for the set of municipalities participating in the study. Literacy of the population of 15 years or older reflects on the socioeconomic conditions of families and results in improved quality of health care. The high educational level is attributed to the ability to acquire knowledge in health matters and the optimized use of primary care services and consequently better health outcomes [82, 88].

The multivariate regression models (Tables 4 and 5) used to evaluate the morbidity outcomes due to malnutrition and diarrhea, respectively, highlight the excellent contribution of the PBF [72, 89], present with higher coverage of care of the population in poorer municipalities, which are in turn responsible for higher average morbidity rates due to these diseases. Similarly, for the set of municipalities evaluated, those presenting higher coverage of access to water and higher average morbidity rates due to these causes, the explanation is a reflection of better municipal and hospital structure of municipalities that have higher coverage of access to water, making them more able to receive severe cases of these diseases and thus, subject to hospitalization [90]. Another explanation for these high mean morbidity rates, especially for the diarrhea outcome, in models where the urbanization rate negatively affected the outcome, is the presence in these municipalities with high coverage of access to water, a significant portion of the population residing in peri-urban areas [81, 82]. At the same time, other variables of environmental health, access to sanitation and solid waste collection, were associated with a decrease in the average rates of morbidity due to malnutrition and diarrhea as access coverage to these services increases, which reinforces the need for greater investments, mainly related to sanitation, in the peripheral areas of urban centers and in rural areas, where the poorest population is concentrated and which suffers greater health impacts due to the absence of adequate environmental health structures [91, 92].

Finally, for the interaction models (Tables 7 and 8), that evaluated the outcomes malnutrition and diarrhea, the results indicate that when the two public policies were included in the same equation, the presence of one (environmental health interventions related to access to solid waste collection and sanitation) did not positively modify the action of the other (total PBF), resulting in the expected decrease in the average morbidity rates for these diseases. This result suggests that only the inclusion of these indicators was not sufficient to change the health conditions of municipalities that present high percentages of the population with high economic and social vulnerability. However, the variables sanitation and solid waste modified the effect of the water variable, resulting in protective environments when present simultaneously in the municipalities, as well as protective environments in the presence of adequate conditions of access to sanitation and collection of solid waste concomitantly.

Thus, in light of the above, the interaction models used were able to show that the simultaneous presence of high municipal coverage of access to adequate sanitation services, collection of solid waste and access to water results in a decrease in the average morbidity rates due to malnutrition and diarrhea. Regardless of whether the municipality is poor or consequently covered by the PBF, the presence of high coverage of environmental health variables results in a decrease in the processes of illness due to malnutrition and diarrhea in children under five years of age. The results provide evidence that a combination of interventions related to environmental health (access to water, sanitation and solid waste collection) may be necessary to generate a significant impact on health outcomes, especially in municipalities that have high values of water coverage, but without adequate sanitation coverage and solid waste collection.

The limitations of the current study are the need to use statistical methods of interpolation and extrapolation for estimating the values of some independent variables and the use of hospitalization data, from the Hospital Information System (SIH), to evaluate morbidity processes, which refer only to hospitalization data by the Unified Health System (SUS). However, in relation to interpolated and extrapolated data, any potential bias, which could have caused a decrease in the real fluctuations of the measures over the years, was minimized by comparing the interpolated and extrapolated data with the real data collected through PNADs [93] and SNIS [94], for the years 2006 to 2009 and 2011 to 2016, and verifying compatible measures for these values.

Strengths of the study that validate its results are: (i) selection only of municipalities that presented adequacy of vital statistics data, which guarantees its internal validity; (ii) the possibility, through the Generalized Linear Model with the Negative Binomial distribution of fixed effects without and with zero inflation, of evaluating all 3,467 participating municipalities (62.35% of the universe of Brazilian municipalities), which presented as a characteristic the excess of zeros of the response variables (70.82% of the malnutrition outcome and 14.11% of the diarrhea outcome); (iii) use of ecological data at the municipal level that allows for the evaluation of large populations, to cover divergent population groups in relation to exposure, the ease and low cost of obtaining the data, as well as the increasing availability of large databases that facilitate the aggregation of numerous variables and the possibility of more comprehensively evaluating the socioeconomic, political and environmental determinants involved in the health-disease process through this type of study [95–97].

Based on the results obtained, it is concluded that although there were important advances in terms of the decrease in hospitalization rates due to malnutrition and diarrhea in the period evaluated, a significant rate is still observed in relation to morbidity due to diarrhea in children less than five years of age, which should not be disregarded. The multivariate models with and without interaction showed that specific actions may not have immediate positive effects and the concomitant presence of actions that present complementary objectives can revert inadequate situations and provide safer environments for the population. Thus, our suggestions include the expansion and maintenance of the coverage of the population eligible for the PBF and emergency assistance, and the universalization of environmental health services, mainly related to the coverage of sanitation and solid waste collection.

## Abbreviations

AGVIF: generalized variance inflation factors
AIC: Akaike Information Criterion
BIC: Bayesian Information Criterion
CID-10: 2International Statistical Classification Codes for Diseases and Health-Related Problems—10^th^ revision
DATASUS: Department of Informatics of the Single Health System
DRIS: Diseases Related to Inadequate Environmental Sanitation
ESF: Family Health Strategy
GDP: Gross Domestic Product
GVIF: generalized variance inflation factors
IBGE: Brazilian Institute of Geography and Statistics
IPCA: Broad Consumer Price Index
IRR: Ratio for Incidence Rates
MIS: Social Information Matrix
PBF: Bolsa Família Program
PLANSAB: Basic National Sanitation Plan
SIH: Hospital Information System
SUS: Unified Health System
